# Glutathione as a Redox Biomarker in Mitochondrial Disease—Implications for Therapy

**DOI:** 10.3390/jcm6050050

**Published:** 2017-05-03

**Authors:** Gregory M. Enns, Tina M. Cowan

**Affiliations:** Departments of Pediatrics and Pathology, Stanford University, 300 Pasteur Drive, H-315, Stanford, CA 94005–5208, USA; tina.cowan@stanford.edu

**Keywords:** mitochondrial disease, glutathione, redox imbalance, EPI-743, *N*-acetylcysteine, RP103, cysteamine

## Abstract

Technical advances in the ability to measure mitochondrial dysfunction are providing new insights into mitochondrial disease pathogenesis, along with new tools to objectively evaluate the clinical status of mitochondrial disease patients. Glutathione (l-ϒ-glutamyl-l-cysteinylglycine) is the most abundant intracellular thiol, and the intracellular redox state, as reflected by levels of oxidized (GSSG) and reduced (GSH) glutathione, as well as the GSH/GSSG ratio, is considered to be an important indication of cellular health. The ability to quantify mitochondrial dysfunction in an affected patient will not only help with routine care, but also improve rational clinical trial design aimed at developing new therapies. Indeed, because multiple disorders have been associated with either primary or secondary deficiency of the mitochondrial electron transport chain and redox imbalance, developing mitochondrial therapies that have the potential to improve the intracellular glutathione status has been a focus of several clinical trials over the past few years. This review will also discuss potential therapies to increase intracellular glutathione with a focus on EPI-743 (α-tocotrienol quinone), a compound that appears to have the ability to modulate the activity of oxidoreductases, in particular NAD(P)H:quinone oxidoreductase 1.

## 1. Introduction

“Do you feel any better?” is a commonly asked question by a physician caring for a patient who has an underlying mitochondrial disorder during a clinic visit, typically after an interval of time following the start of various co-factors, vitamins, or supplements that may have a beneficial effect on mitochondrial function [1]. The lack of validated, widely available, and objective markers of mitochondrial function makes this state-of-the-art of mitochondrial medicine in the 21st century somewhat discouraging. 

Nevertheless, there have been clear advances in our ability to determine clinical severity in mitochondrial disease patients. Clinical scoring tools, especially the Newcastle Mitochondrial Disease Adult Scale (NMDAS) and the Newcastle Paediatric Mitochondrial Disease Scale (NPMDS), provide the means to quantify the clinical burden of mitochondrial disease in individual patients [2,3]. Improvements in the resolution of traditional imaging techniques (e.g., magnetic resonance imaging and magnetic resonance spectroscopy) and the emergence of complementary new methods are also encouraging [4,5,6,7]. Finally, recent discoveries of minimally-invasive mitochondrial biomarkers, including blood creatine, FGF-21, and GDF-15, provide increased sensitivity and specificity compared to the standard practice of measuring lactate [8,9,10,11], although the link between these new biomarkers and mitochondrial disease pathophysiology is still unclear. 

In contrast to these new analytes, which were detected by global metabolomic or transcriptomic profiling [9,12,13], there is a clear theoretical link between pathophysiology and biomarkers that are directly related to the biochemistry of redox imbalance. One important example is glutathione: mitochondrial dysfunction has been implicated in the generation of increased reactive oxygen and nitrogen species (RONS) leading, in turn, to increasing redox imbalance and decreased reduced glutathione (GSH) levels. Of course, the utility of this, and other, biomarkers still needs to be established in clinical practice. However, with improvements in both our understanding of mitochondrial disease pathogenesis and technologies to measure mitochondrial dysfunction, we may be approaching a time when clinicians will have quantitative, validated measures to gauge the clinical status of their mitochondrial disease patients. The ability to quantify mitochondrial dysfunction in an affected patient will not only help with routine care, but also improve rational clinical trial design aimed at developing new therapies.

## 2. Glutathione Levels in Mitochondrial Disorders

Glutathione (l-ϒ-glutamyl-l-cysteinylglycine) is the most abundant intracellular thiol, with intracellular concentrations ranging from about 0.5 to 10 μM. In its reduced form (GSH), it plays a key role in cellular free radical defense [14,15]. Glutathione synthesis occurs in the cytosol with 85% to 90% of GSH localized to the cytoplasm. The remainder is distributed between various organelles, including peroxisomes, the nuclear matrix, endoplasmic reticulum, and mitochondria [16,17,18]. 

The intracellular redox state, as reflected by levels of oxidized (GSSG) and reduced (GSH) glutathione, as well as the GSH/GSSG ratio, is an important indicator of cellular health [19,20]. By evaluating levels of GSH and GSSG, as well as the GSH/GSSG ratio in blood, one can get a glimpse into the degree of mitochondrial dysfunction at a tissue level as these compounds are leaked into the surrounding blood stream, urine or cerebrospinal fluid [8]. The GSSG/2GSH redox couple is representative of the redox environment in an individual, because the glutathione system plays a central role in maintaining the overall redox status of the body [19,21]. Dysfunction of the mitochondrial electron transport chain is associated with redox imbalance and abnormally low GSH levels in primary genetic mitochondrial disorders, as well as conditions associated with secondary mitochondrial impairment, such as organic acidemias, Friedreich ataxia, Alzheimer disease, Parkinson disease, amyotropic lateral sclerosis, and Rett syndrome [22,23,24,25,26,27,28,29]. Table 1 shows examples of glutathione determination by various methods in mitochondrial disorder and organic acidemia patients. Indeed, the documented redox abnormalities in patients who have either primary or secondary mitochondrial dysfunction have led to the development of mitochondrial therapies that have the potential to improve intracellular glutathione status [30]. 

While the glutathione redox couple represents an attractive mitochondrial biomarker, measured concentrations of GSH and GSSG have varied between different laboratories, likely because of instability of GSH during specimen handling and the use of various analytical methods, including high-performance liquid chromatography (HPLC), gas chromatography with mass spectrometry, capillary electrophoresis with ultraviolet absorbance or colormetric detection, and liquid chromatography-tandem mass spectrometry (LC-MS/MS) [31]. GSSG levels are especially influenced by oxidation during sample handling, and can appear elevated if conditions are not properly controlled [32,33,34]. Biological samples in which GSH, GSSG, and GSH/GSSG have been determined include whole blood, plasma, erythrocytes, leukocytes, urine, and skeletal muscle [24,31,35,36,37,38,39,40,41,42,43,44].

Increased plasma lipid peroxidation and decreased plasma and erythrocyte GSH levels were detected using an HPLC method in 11 patients with chronic progressive external ophthalmoplegia (CPEO) and muscle biopsies with ragged-red fibers and scattered cytochrome *c* oxidase (COX) deficiency [43]. Skeletal muscle biopsies from 17 patients with CPEO, MELAS, or myoclonic epilepsy with ragged-red fibers (MERRF) showed induction of the antioxidant enzymes manganese and copper-zinc superoxide dismutase in fibers associated with ETC deficiency; GSH was found to be elevated in these fibers by histochemical analysis. Antioxidants were expressed in both ragged-red fibers and fibers with subsarcolemmal mitochondrial accumulations that were COX negative. The authors concluded that increased GSH represented the earliest defense against the toxic effects of ETC-produced hydrogen peroxide [44]. 

HPLC analysis of 24 skeletal muscle biopsies from mitochondrial disease patients with defined ETC defects showed a significant decrease in GSH concentration compared to 15 age-matched controls without evidence of mitochondrial ETC deficiency (7.7 ± 0.9 nmol/mg protein vs. 12.3 ± 0.6 nmol/mg protein). Furthermore, the most prominent GSH deficiency was noted in the patients who had multiple ETC defects in complexes I, II − III + IV [24]. The authors postulated that treatments designed to increase GSH levels, such as *N*-acetylcysteine or oxothiazolidine-4-carboxylate supplementation, may be beneficial to patients who have ETC deficiency associated with a GSH deficit [24]. 

Intracellular leukocyte GSH levels were evaluated in blood samples from patients with either mitochondrial diseases or organic acidemias using high-dimensional flow cytometry (Hi-D FACS). T lymphocyte subsets, monocytes, and neutrophils showed low GSH levels in both mitochondrial disease and organic acidemia patients, although levels were relatively normal in those patients who were taking antioxidants [25]. The Hi-D FACS results demonstrated redox imbalance in patients with either primary or secondary mitochondrial dysfunction, but the technique is semi-quantitative and not wholly amenable to the clinical setting, as samples have to be analyzed immediately and cannot be shipped. 

A study of 10 mitochondrial disease patients with a variety of clinical presentations, including Leigh syndrome, Alpers syndrome, Kearns–Sayre syndrome, and multisystem disease analyzed plasma GSH and cysteine levels by HPLC. Plasma GSH levels were low in mitochondrial disease patients, mostly below the detection level of the method used, and reduced cysteine levels were also lower in mitochondrial disease patients compared to controls. Erythrocyte thiols and glutathione-related enzymes, such as glutathione peroxidase, glutathione reductase, and glutathione S-transferase, were also evaluated, but significant differences between patients and controls were not observed [41]. 

LC-MS/MS appears to be particularly promising as a methodology for analyzing glutathione samples. Whole blood samples can be deproteinized with sulfosalicylic acid and derivatized with *N*-ethylmaleimide (NEM) in order to prevent oxidation of GSH in a single step before being analyzed. Derivatized samples are stable for at least three years when stored at −80 °C, and underivatized samples for at least 24 h at room temperature, allowing potential implementation in clinical laboratories [31]. This LC-MS/MS method was initially used to study healthy individuals and mean ± SD glutathione levels were: GSH 900 ± 140 μM; GSSG 1.17 ± 0.43 μM; and GSH/GSSG 880 ± 370 [31]. 

A further study used LC-MS/MS to measure whole blood glutathione levels in mitochondrial disease patients with a variety of different clinical phenotypes, including Leigh syndrome, mitochondrial encephalomyopathy, lactic acidosis and stroke-like episodes (MELAS), mtDNA deletion syndrome, conditions associated with mtDNA depletion, and patients with a variety of electron transport disorders. A subset of patients was evaluated both while in times of relative good health and while hospitalized during a metabolic crisis. Compared to healthy controls, mitochondrial disease patients (*n* = 58), as a whole, showed significantly lower whole blood GSH levels (808 ± 149 μM vs. 900 ± 141 μM, *p* = 0.0008), GSH/GSSG ratio (881 ± 374 vs. 596 ± 424, *p* = 0.0002), and higher GSSG levels (2.23 ± 1.84 μM vs. 1.17 ± 0.43 μM, *p* < 0.0001) [28]. In addition to measuring absolute levels of GSH and GSSG, whole blood redox potential was calculated using the Nernst equation. Mitochondrial disease patients had significant redox imbalance, with an increased degree of oxidation of approximately 9 mV compared to controls (−251 ± 9.7 mV vs. −260 ± 6.4 mV). When subgroups of mitochondrial disease patients were evaluated, redox potential was significantly more oxidized in each group. Interestingly, the lowest GSH levels in relatively healthy patients were found in those with Leigh syndrome (735 ± 135 μM). Overall, patients who were hospitalized for treatment of a metabolic crisis showed the lowest GSH levels (550 ± 93 μM) and the greatest degree of redox imbalance (−242 ± 7.0 mV) [28].

In aggregate, there is clear evidence of GSH deficiency and redox imbalance in mitochondrial disease patients. However, further longitudinal studies are needed to determine the utility of using the glutathione system as a biomarker of disease severity and response to therapies.

## 3. Glutathione Levels in Other Disorders Associated with Mitochondrial Dysfunction

### 3.1. Organic Acidemias

Abnormal mitochondrial structure and function, as well as various abnormal measures of oxidative stress and redox imbalance, have been reported in animal models and patients affected by a variety of organic acidemias, including methylmalonic acidemia, cobalamin A disease, cobalamin C disease, cobalamin H/cobalamin D disease, propionic acidemia, isovaleric acidemia, 2-methyl-3-hydroxybutyric acidemia, 3-methylglutaconic acidemia types II and IV, d-2-hydroxyglutaric aciduria, l-2-hydroxyglutaric aciduria, and glutaric acidemia [42,45,46,47,48,49,50,51,52,53,54,55,56,57,58,59,60,61,62,63,64,65,66,67,68,69,70]. GSH levels have been found to be low in methylmalonic acidemia (MMA), propionic acidemia (PA), and isovaleric acidemia (IVA) [25,26,71]. A seven-year-old boy with *mut^−^* methylmalonic acidemia in metabolic crisis was found to have marked lactic acidemia, GSH deficiency, and 5-oxoprolinuria; treatment with ascorbate, in addition to other supportive management, resulted in an improvement in clinical status and resolution of the biochemical abnormalities [71]. Hi-D FACS analysis of peripheral blood leukocytes was performed in 13 patients with MMA, PA, or IVA. Organic acidemia patients who had samples collected during an illness severe enough to require hospitalization showed significantly lower intracellular GSH levels in CD4 T cells, CD8 T cells, monocytes, and neutrophils when compared to healthy controls. On the other hand, patients who had samples collected during routine outpatient visits had lower GSH levels detected only in CD4 and CD8 T cells [25]. A more recent study used an HPLC method to evaluate GSH levels in 11 MMA, PA, and IVA patients and showed that organic acidemia patients had lower plasma GSH levels than controls. Organic acidemia patients also had a greater fraction of GSH and cysteine in an oxidized state [41].

### 3.2. Friedreich Ataxia

Friedreich ataxia patients also have evidence of redox abnormalities and mitochondrial dysfunction [72,73,74,75]. A study of 14 unrelated Friedreich ataxia patients measured total and free GSH concentrations in erythrocytes by HPLC. Patients had a significant reduction of free glutathione levels, although total glutathione levels were comparable to controls. Friedreich ataxia patients were also found to have a significant increase in glutathione bound to hemoglobin in erythrocytes [23]. Glutathione homeostasis was, therefore, considered to be impaired in Friedreich ataxia, raising the possibility that free radicals play a role in disease pathophysiology [23].

### 3.3. Parkinson Disease and Other Neurodegenerative Disorders

Mitochondrial dysfunction and oxidative stress also appear to be central to disease pathogenesis in Parkinson disease and other neurodegenerative conditions, including Alzheimer disease, amyotropic lateral sclerosis, and Rett syndrome [27,76,77,78,79,80,81]. Not surprisingly, the glutathione axis has been found to be abnormal when evaluated in these conditions [20,27,82,83]. As reviewed elsewhere in this issue, children with autistic spectrum disorder have also been found to have biochemical abnormalities suggestive of an underlying impaired mitochondrial metabolism, including low levels of GSH and low GSH/GSSG ratio [84,85,86].

### 3.4. Genetic Syndromes

Finally, redox and GSH abnormalities have been identified in a number of genetic syndromes, including Down syndrome, Werner syndrome, fragile X syndrome, and Kindler syndrome [87,88,89,90,91]. Therefore, it is possible that mitochondrial dysfunction may play some role in the clinical findings associated with conditions that appear to have no or a minimal relationship to mitochondrial metabolism. Further studies are clearly needed in order to determine the significance of these initial reports. 

## 4. Implications for Mitochondrial Disease Therapy

Since multiple disorders have been associated with either primary or secondary deficiency of the mitochondrial ETC and the resultant redox imbalance, developing mitochondrial therapies that have the potential to improve the intracellular glutathione status has been a focus of several clinical trials over the past few years [92,93,94,95,96,97,98,99,100,101,102]. For example, EPI-743 (α-tocotrienol quinone) is an investigational drug that is currently in clinical trials focusing on treatment of mitochondrial dysfunction related to primary genetic mitochondrial disease, including Leigh syndrome, Leber Hereditary Optic Neuropathy (LHON), and RARS2 deficiency. EPI-743 is also being used in other conditions that have redox imbalance linked to disease pathophysiology, including Friedreich ataxia, Parkinson disease, and Rett syndrome (Table 2). 

## 5. EPI-743

EPI-743 is a *para*-benzoquinone analog that is approximately one thousand- to ten thousand-fold more potent than coenzyme Q_10_ or idebenone in protecting mitochondrial patient fibroblasts when a strong oxidant stress is applied [103]. This beneficial effect is considered to be related to the ability of EPI-743 to modulate the activity of oxidoreductases, in particular NAD(P)H:quinone oxidoreductase 1, resulting in increased cellular GSH concentration and improvement in redox status [30,92,103]. EPI-743 may also affect antioxidant gene expression, as pre-treatment of fibroblasts derived from a polymerase γ deficiency patient with EPI-743 before exposure of cells to oxidative stress resulted in a blunting of antioxidant response element (ARE) gene expression in genes under direct control of nuclear factor-erythroid 2 p45-related factor (Nrf2), including genes related to GSH synthesis [92]. 

### 5.1. Mitochondrial Disorders

The initial experience with EPI-743 in mitochondrial disease was reported in 13 children and one adult enrolled in a 13-week emergency treatment protocol for patients who were considered to be at risk for progressing to end-of-life care within 90 days by experienced clinicians. Surviving patients were then placed in an extension protocol. Twelve of the 14 patients survived during the period of observation; 11 of the survivors demonstrated clinical improvement, with three showing partial relapse. NPMDS scores were not significantly different for sections I, II, or III (sections related to clinical status) when scores from before and after EPI-743 treatment were compared, whereas 10 patients showed improvement in section IV (quality of life) [92]. 

Twelve of the 14 patients also underwent serial brain imaging using technetium-99m-hexamethylpropyleneamine oxime (HMPAO) SPECT. HMPAO is a lipophilic radionuclide tracer that is sensitive to intracellular redox status that is used to measure cerebral blood flow, as well as intracellular GSH and reduced protein thiols. This tracer is retained inside cells, locked in a hydrophilic state, in the presence of adequate reducing equivalents generated by functional mitochondria [104]. Before the administration of EPI-743, all 12 individuals had decreased brain HMPAO uptake compared to normal controls. After three months of EPI-743 therapy, there was a significant increase in whole brain HMPAO uptake [92]. A further study of 22 patients enrolled in the EPI-743 emergency treatment protocol demonstrated an increase in HMPAO uptake in the cerebellum in all patients. Furthermore, there was a significant correlation between increased cerebellar uptake and improved Newcastle score (*r* = 0.623; *p* = 0.00161). The subgroup of five patients with MELAS showed a significant relationship between whole brain HMPAO uptake and Newcastle score improvement (*r* = 0.917; *p* = 0.028) [7].

### 5.2. Leber Hereditary Optic Neuropathy

EPI-743 has also been used to treat LHON patients. An open-label trial using EPI-743 was performed in five LHON patients, including a child harboring the m.14484T > C variant (associated with spontaneous recovery in some cases), three patients with the m.11778G > A variant and one with the m.3460G > A variant. EPI-743 arrested disease progression and reversed vision loss in all but one of these consecutively-treated patients [93]. 

An open-label study of EPI-743 therapy in ten children with genetically-confirmed Leigh syndrome showed stabilization and even reversal of disease progression. A significant improvement was noted for each of the primary outcome endpoints, which included the NPMDS, and measures of gross motor function, and quality of life (*p* < 0.05) [94]. Enrolled subjects also had total, reduced, and protein-bound glutathione levels measured in lymphocytes before and after treatment with EPI-743. At baseline, the Leigh syndrome subjects had decreased total and reduced glutathione levels, as well as high levels of oxidized glutathione. Following treatment with EPI-743 a marked increase in reduced glutathione (*p* < 0.001) and a 96% decrease in the ratio of oxidized-to-reduced glutathione (*p* < 0.001) was observed [30].

### 5.3. Leigh Syndrome

EPI-743 is currently being used in a randomized, double blind, placebo-controlled clinical trial in children with Leigh syndrome (NCT01721733; NCT02352896). Clinical trial design included a six-month placebo-controlled phase, followed by a 30-month extension phase to assess long-term drug safety and impact on disease morbidity. In the initial six-month phase, treatment with EPI-743 was associated with fewer subjects requiring hospitalization or experiencing serious adverse events as compared with those subjects who received a placebo (11.8% vs. 42.8%). Further follow-up of enrolled subjects indicated that there was a progressive decline in hospitalizations and serious adverse events from the first six months of EPI-743 treatment to months 19 to 24 [95].

### 5.4. RARS2 Deficiency

Autosomal recessive pathogenic variants in *RARS2*, the gene encoding mitochondrial arginyl-transfer RNA synthetase, have been associated with an early-onset mitochondrial encephalopathy characterized by microcephaly, profound developmental delay, intractable seizures, dystonia, and pontocerebellar hypoplasia [96]. In an open-label study, five children with RARS2 deficiency were given EPI-743 over a 12-month treatment phase, followed by an extension phase that is still ongoing. All subjects demonstrated an improvement in clinical status regardless of the severity of baseline disease. Status epilepticus resolved in two children, and the other three children demonstrated a reduction in seizure frequency and duration [97]. 

### 5.5. Friedreich Ataxia

In a study of EPI-A0001, an α-tocopheryl quinone structurally related to EPI-743, 31 adults with Friedreich ataxia were evaluated using a measure of diabetic tendency as the primary clinical trial outcome measure (NCT01035671). The Friedreich Ataxia Rating Scale (FARS) was used as a secondary neurological outcome measure [105]. No significant difference was observed in the measure of diabetic tendency between treated subjects and controls after four weeks of therapy. However, a dose-dependent improvement in the FARS score was observed, indicating that this compound potentially has an effect on the central nervous system [98]. 

In a phase 2 double-blind placebo-controlled trial of EPI-743 in adults with Friedreich ataxia (NCT01728064), EPI-743 treatment resulted in a significant improvement in neurological function and disease progression when compared to controls as measured by the FARS. The improvement in FARS score was dose-dependent; subjects who received EPI-743 at the highest dosage for the entire 24-month study period registered the greatest degree of improvement [99].

Friedreich ataxia may rarely be caused by a point mutation in *FXN* on one allele in combination with a typical GAA trinucleotide repeat expansion on the other allele. Four Friedreich ataxia patients who harbor a point mutation in one *FXN* allele were treated with EPI-743 in an open-label study (NCT01962363). The patients showed clinical improvement as assessed by FARS score over 18 months of therapy [100].

### 5.6. Rett Syndrome

In a six-month, randomized, double-blind, placebo-controlled trial involving 24 Rett syndrome patients aged 2.5–8 years (NCT01822249), those who were treated with EPI-743 showed a significant increase in head circumference relative to placebo subjects (*p* = 0.05). In a subgroup of children with the greatest degree of head growth, improvements in oxygenation, hand function, and disease biomarkers were also observed [101].

### 5.7. Parkinson Disease

A phase 2a open-label pilot study was performed to determine if EPI-743 might improve the treatment of Parkinson disease (NCT01923584). The Unified Parkinson Disease Rating Scale (UPDRS) [106], electroretinography, and brain metabolite levels as measured by magnetic resonance spectroscopy (MRS) were used as clinical outcome measures. Six of seven patients with follow-up MRS studies showed a decrease in glutamine/glutamate levels in the basal ganglia opposite the side most severely affected by Parkinson disease. In addition, improvement in retinal function was noted on evaluation by electroretinogram. Subjects also demonstrated an improvement in UPDRS scores that approached statistical significance [102]. 

## 6. *N*-Acetylcysteine and Cysteamine

Other compounds that have the potential to increase intracellular GSH include *N*-acetylcysteine (NAC) and cysteamine [107,108]. NAC is a drug that is best known for its therapeutic effects in acetaminophen-induced liver failure [107,109,110], but has also shown promise in acute liver failure in the absence of acetaminophen overdose [110,111]. Since oxidative and nitrosative stress play a role in the pathogenesis of liver failure and the subsequent CNS effects of hepatic encephalopathy [112,113], the replenishment of intracellular GSH by NAC may be beneficial [107]. NAC has also been used as a mucolytic for the treatment of cystic fibrosis, and to treat chronic obstructive pulmonary disease, diabetes mellitus, and patients infected with human immunodeficiency virus [107]. 

NAC has been shown to improve markers of oxidative stress in an animal model of Huntington disease and cell lines derived from patients with Huntington disease and mitochondrial respiratory chain disorders [114,115,116]. Although there have been case reports using NAC to treat primary mitochondrial disorders, for example, in mitochondrial disease patients who have liver dysfunction [117], to our knowledge there have not yet been controlled clinical trials that explore the efficacy of NAC in these conditions. On the other hand, NAC has been used in controlled trials in several conditions with likely secondary mitochondrial involvement, including Alzheimer disease, amyotropic lateral sclerosis, and autism [118]. Improvement in some measures of cognitive ability was observed in Alzheimer disease patients, but no improvement in survival or disease progression was noted in those with amyotropic lateral sclerosis. Autistic patients have shown improvement in some aberrant behaviors, especially irritability, following treatment with NAC [118]. 

NAC has also been used in combination with metronidazole to treat ethylmalonic encephalopathy, a disorder caused by mutations in *ETHE1* that result in secondary inhibition of cytochrome *c* oxidase and other enzymes. Treated *Ethe1*-deficient mice had a prolonged lifespan, and five ethylmalonic encephalopathy patients demonstrated marked clinical improvement following combined therapy [119]. 

Cysteamine, an established therapy for cystinosis, serves to decrease the abnormal lysosomal storage of cystine. Cysteamine also appears to promote the transport of cysteine into cells, which could increase intracellular glutathione levels [108]. Cysteamine has been shown to improve symptoms in mouse models of Huntington disease, and has been used in a small open-label clinical trial in Huntington disease patients [120]. Clinical efficacy was not demonstrated, although the trial established a safe cysteamine dosage regimen in Huntington disease patients [120,121]. Cysteamine bitartrate delayed-release (RP103) is a microsphere formulation associated with decreased gastrointestinal symptoms [122]. RP103 is currently being used in a randomized, controlled, double-blind multicenter trial for Huntington disease (NCT02101957) and an open-label study in children with mitochondrial disease (NCT02023866), but results have not yet been published. 

In summary, the glutathione system continues to be evaluated as a potentially valuable biomarker of mitochondrial dysfunction across multiple diseases. There have been clear advances in the field of mitochondrial redox biomarker analysis, with glutathione levels being able to be measured by improved analytical techniques in virtually any tissue sample, as well as by using relatively non-invasive brain imaging techniques, such as HMPAO SPECT. Measuring of glutathione metabolites has provided investigators with unique insights into the redox imbalance present in patients who have mitochondrial dysfunction, which has led to clinical trials designed to address this issue. In the near future, clinicians caring for individuals affected by mitochondrial disease may not only have improved therapies to offer their patients, but may also be able to monitor individuals by blood and imaging biomarkers. By doing so, physicians may be able to both predict and understand in advance the answer to the question, “Do you feel any better?”

## Figures and Tables

**Table 1:** Glutathione status in mitochondrial disorders and organic acidemias.

Conditions	Age	Analytical Method	Results	Reference
CPEO (*n* = 11)	30–70 years	HPLC	Plasma GSH: 8.64 ± 1.82 μM (controls 11 ± 3 μM; *p* < 0.01) RBC-GSH: 12.41 ± 2.37 nmol/mg protein (controls 18 ± 2.2 nmol/mg protein)	[43]
CPEO (*n* = 14) MELAS (*n* = 2) MERRF (*n* = 1)	19–86 years	GSH histo-chemistry	Muscle GSH: Induction of GSH in fibers with respiratory chain deficiency	[44]
Mitochondrial disorders (*n* = 24)	1 month–12 years	HPLC	Muscle GSH: Complexes I, II − III + IV (*n* = 7) 4.1 ± 0.98 nmol/mg protein (*p* < 0.05) Complex I (*n* = 1) 4.9 nmol/mg protein Complexes II − III + IV (*n* = 1) 3.0 nmol/mg protein Complex IV (*n* = 11) 9.7 ± 1.39 nmol/mg protein Complex I + IV (*n* = 7) 10.3 ± 1.80 nmol/mg protein Controls (*n* = 15) 12.3 ± 0.62 nmol/mg protein	[24]
Mitochondrial disorders (*n* = 20)	3–36 years	Hi-D FACS	Leukocyte GSH: Decreased iGSH in CD4 T cells (*p* = 0.014), CD8 T cells (*p* = 0.005), monocytes (*p* = 0.016), and neutrophils (*p* = 0.044)	[25]
Mitochondrial disorders (*n* = 10)	4–14 years	HPLC	Plasma GSH: 26.3 μM (controls 48.9 μM; *p* = 0.031) RBC-GSH: 6.4 ± 1.1 μmol/g protein (controls 6.7 ± 0.56 μmol/g protein)	[41]
Mitochondrial disorders (*n* = 58)	6 months–50 years	LC-MS/MS	Whole blood GSH: 808 ± 149 μM (controls 900 ± 140 μM; *p* = 0.0008) Whole blood GSSG: 2.23 ± 1.84 μM (controls 1.17 ± 0.43 μM; <0.0001) Whole blood GSH/GSSG: 596 ± 93 μM (controls 800 ± 370 μM; *p* = 0.0002) Whole blood redox potential: −251 ± 9.7 mV (controls −260 ± 6.4 mV)	[28,31]
Friedreich ataxia (*n* = 14)	8–22 years	HPLC	Whole blood GSH + GSSG: 0.55 nmol/mg hemoglobin (controls 8.4 ± 1.79 nmol/mg hemoglobin; *p* < 0.001) RBC hemoglobin-bound glutathione: 15 ± 1.5% (controls 8 ± 1.8%; *p* < 0.05)	[23]
Organic acidemias (*n* = 9)	1 week–6 years	Hi-D FACS	Leukocyte GSH: Decreased iGSH in CD4 T cells (*p* = 0.008), CD8 T cells (*p* = 0.003), monocytes (*p* = 0.0008), and neutrophils (*p* = 0.0006) in hospitalized patients Decreased iGSH in CD4 T cells (0.040) and CD8 T cells (0.045) in outpatients	[25]
Organic acidemias (*n* = 11)	1–16 years	HPLC	Plasma GSH: 32.9 ± 6.9 μM (controls 48.9 ± 25.7 μM)	[26]
Cobalamin C disease (*n* = 18)	1–14 years	HPLC	Lymphocyte total glutathione: 23 nmol/mg protein (95% CI 10.25–62.03) (controls 6.9 nmol/mg protein; 95% CI 21.96–60.42; *p* < 0.05) Lymphocyte GSH: 6.9 nmol/mg protein (95% CI 0.68–24.83) (controls 39.10 nmol/mg protein; 95% CI 19.31–54.55; *p* < 0.001) Lymphocyte GSSG: 7.9 nmol/mg protein (95% CI 1.87–24.78) (controls 2.94 nmol/mg protein; 95% CI 1.33–3.82; *p* < 0.05)	[42]

Note: The glutathione status as determined by different analytical methodologies in mitochondrial disease or organic acidemia patients is shown above. Statistical significance is shown where possible as provided by the individual references. CI = confidence interval; CPEO = chronic progressive external ophthalmoplegia; GSH = reduced glutathione; GSSG = glutathione disulfide; HiD-FACS = high-dimensional fluorescence-activated cell sorting; HPLC = high-performance liquid chromatography; iGSH = intracellular reduced glutathione; LC-MS/MS = liquid chromatography-tandem mass spectrometry; RBC = red blood cell.

**Table 2:** EPI-743 clinical trials.

Patient Population	Age	Trial Design	Duration	Outcomes	Reference
Mitochondrial disease (*n* = 14)	2–27 years	Open-label	98–444 days	11/12 survivors with clinical improvement; 3/11 partial relapse; 10/12 improvement in quality of life (NPMDS section IV); 2 deaths	[92]
LHON (*n* = 5)	8–52 years	Open-label	204–557 days	4/5 arrested disease progression and reversal of vision loss; 2/5 total recovery of visual acuity	[93]
Leigh syndrome (*n* = 10)	1–13 years	Open-label	6 months	Reversal of disease progression; Improvement in NPMDS, GMFM, PedsQL Neuromuscular Module (*p* < 0.05)	[94]
Leigh syndrome (*n* = 35)	9 months–14 years	Randomized, double-blind, placebo-controlled	36 months	Decreased rate of hospitalization and serious adverse events	[95]
RARS2 deficiency (*n* = 5)	5–13 years	Open-label	1 year	Improved neuromuscular function and redox state; Decreased seizure frequency with 2 patients showing resolution of status epilepticus	[96,97]
Friedreich ataxia (*n* = 31) ^1^	18–66 years	Randomized, double-blind, placebo-controlled	28 days	Dose-dependent improvement in FARS score; No alteration in Disposition Index (measure of diabetic tendency)	[98]
Friedreich ataxia (*n* = 63)	19–43 years	Randomized, double-blind, placebo-controlled	2 years	Dose-dependent improvement in FARS score	[99]
Freidreich ataxia (point mutations) (*n* = 4)	21–63 years	Open-label	18 months	Improvement in FARS	[100]
Rett syndrome (*n* = 24)	2.5–8 years	Open-label	6 months	Primary endpoint of improvement in Rett syndrome disease severity score not met; Increase in head circumference (*p* = 0.05); Improved oxygenation, hand function and disease biomarkers in subgroup with greatest degree of head growth	[101]
Parkinson disease (*n* = 10)	43–69 years	Open-label	6 months	Improvement in UPDRS Parts II/III; Decrease in brain glutamine/glutamate levels; Improvement of retinal function on electroretinogram	[102]

^1^ EPI-A0001 was used in this study, not EPI-743. EPI-A0001 is an α-tocopheryl quinone drug with a chemical structure similar to EPI-743. FARS = Friedreich Ataxia Rating Scale; GMFM = Gross Motor Function Measure; NPMDS = Newcastle Paediatric Mitochondrial Disease Scale; PedsQL = Pediatrics Quality of Life Inventory; UPDRS = Unified Parkinson Disease Rating Scale.
